# Translation, Cross-Cultural Adaptation and Validation of the Serbian Version of the Clinical Frailty Scale in Patients Undergoing Major Uro-Oncological Surgery

**DOI:** 10.3390/healthcare14111567

**Published:** 2026-06-03

**Authors:** Natasa Petrovic, Nebojsa Ladjevic, Vesna Jovanovic, Dimitrije Sarac, Ana Mimic, Milan Radovanovic, Mila Milicevic, Milos Lazic, Sandra Sipetic Grujicic

**Affiliations:** 1 Center for Anesthesiology and Resuscitation, University Clinical Centre of Serbia, Pasterova 2 St., 11000 Belgrade, Serbia; nladjevic@yahoo.com (N.L.); vantonijevicjov@gmail.com (V.J.); milamilamila94@gmail.com (M.M.); m.lazic1003@gmail.com (M.L.); 2 Faculty of Medicine, University of Belgrade, Dr Subotica 8 St., 11000 Belgrade, Serbia; milan950@hotmail.com; 3 Center for Radiology, University Clinical Centre of Serbia, Pasterova 2 St., 11000 Belgrade, Serbia; dimitrije.sarac89@gmail.com; 4 Royal Free London NHS Foundation Trust, London NW3 2QG, UK; ana.mimic@nhs.net; 5 Clinic of Urology, University Clinical Centre of Serbia, Resavska 51 St., 11000 Belgrade, Serbia; 6 Institute of Epidemiology, Faculty of Medicine, University of Belgrade, Dr Subotica 8 St., 11000 Belgrade, Serbia; sandra.grujicic2014@gmail.com

**Keywords:** clinical frailty scale, frailty, validation, reliability, aging, Serbia, Edmonton frail scale, FRAIL

## Abstract

**Background/Objectives:** Frailty is well-recognized as a predictor of adverse postoperative outcomes. The Clinical Frailty Scale (CFS) is a widely recommended frailty assessment tool due to its simplicity and rapid bedside applicability; however, it has never been validated in Serbia. The aim of this study was to translate, culturally adapt, and validate the CFS in Serbia, in patients undergoing elective major surgical procedures. **Methods:** This cross-sectional study included 149 patients aged ≥50 years undergoing elective major urological oncology surgery. Frailty was assessed preoperatively using three scales: the CFS, the Edmonton Frail Scale (EFS), and the FRAIL scale. The CFS evaluations were independently performed by two raters and repeated after 7 days. Concurrent validity was evaluated via Spearman’s correlation between the CFS, the EFS, and the FRAIL scale. The “known-group” construct validity of the CFS was assessed using the test for trends across clinically relevant groups. Both inter-rater and test–retest reliability were assessed using the intraclass correlation coefficient (ICC). **Results:** The CFS was translated and culturally adapted into the Serbian language in accordance with ISPOR guidelines. The Serbian version of the CFS demonstrated both excellent inter-rater reliability (ICC = 0.957; 95% CI 0.941–0.968), and test–retest reliability (ICC = 0.958; 95% CI 0.943–0.970). A strong positive correlation was observed between the CFS and both the EFS (ρ = 0.698) and the FRAIL scale (ρ = 0.614). A known-group comparison confirmed the construct validity of the CFS. **Conclusions:** The Serbian version of the CFS is a reliable, valid, and clinically feasible tool for preoperative identification of frailty in patients aged 50 years and older undergoing major elective uro-oncological procedures.

## 1. Introduction

Frailty is a complex clinical syndrome characterized by reduced physiological reserve across multiple organ systems, leading to increased vulnerability and decreased ability to manage external stressors, such as acute illness or surgery [1,2]. Traditionally, frailty has been associated with advanced age; however, more recent findings have revealed that frailty can be present at any stage of life [3]. The prevalence of frailty among adults aged 50 years and older is estimated to range from 12% to 24%, with an increasing incidence observed with advancing age, reaching 50% in people 80 years or older. In younger adults, aged 40–64.9 years, the prevalence of frailty is estimated to be approximately 16% [3,4]. This vulnerability is particularly pronounced in oncological populations, in whom the burden of frailty may be greater in comparison with individuals of the same chronological age, but without malignancy. It has been found that cancer and its treatments may contribute to accelerated physiological deterioration, resulting in younger adults with malignancies frequently demonstrating clinical features resembling accelerated biological aging [5].

Frailty is consistently associated with adverse postoperative outcomes. Numerous studies have demonstrated that frail patients are at a significantly higher risk of postoperative complications, including delirium, increased likelihood of institutionalization, prolonged recovery, and both short- and long-term mortality [6]. Among surgical patients, the prevalence of frailty is substantial and varies depending on the type of surgery performed, ranging from approximately 25% to 39% in oncologic surgery, up to 52% in vascular surgery, and between 23% and 59% in orthopedic surgery [7,8,9]. These findings highlight the persistence of frailty in the perioperative population and emphasize the importance of its early identification during preoperative assessment.

Given its strong prognostic significance, a preoperative frailty assessment has been increasingly recommended in recent clinical guidelines as an important component of surgical risk stratification and patient perioperative optimization [10,11]. Nevertheless, despite a growing body of evidence supporting its clinical value, preoperative frailty assessment is still not routinely implemented in everyday surgical practice. A significant challenge to its wider adoption is the lack of validated, linguistically translated, and culturally adapted instruments that can reliably assess frailty across different healthcare settings and populations.

To facilitate the routine use and implementation of frailty assessment in clinical practice, it is essential to ensure that assessment tools are appropriately translated and culturally adapted for the population in which they are used. This adaptation would ensure the conceptual equivalence with the original instruments. The Clinical Frailty Scale (CFS) is one of the most commonly used frailty assessment tools because of its simplicity, rapid bedside applicability, and strong predictive value for postoperative outcomes [1,12,13]. The CFS has been translated into many languages, such as Spanish [14], Brazilian Portuguese [15], Greek [16], Chinese [17], Korean [18], Thai [19], Japanese [20], French [21], Swedish [22], and Danish [23].

Serbian language is the official language in the Republic of Serbia; however, it is widely understood across the South Slavic region, including Croatia, Bosnia and Herzegovina, and Montenegro, and to a lesser degree in Bulgaria, Romania, and North Macedonia. Therefore, the development and validation of a Serbian version of the Clinical Frailty Scale may have relevance beyond the Serbian border, potentially supporting the broader regional implementation of standardized frailty assessment both in clinical and research settings. To date, no formally translated and validated Serbian version of the CFS has been available for Serbian-speaking populations. Creating a validated Serbian version of the CFS would enhance the accessibility of frailty assessment tools for multidisciplinary teams, potentially facilitating a broader implementation of frailty assessments in both clinical and research settings. This is particularly important for perioperative assessments and for patients with chronic and oncological conditions, where early identification of frailty could improve risk stratification, guide patient management, and potentially lead to improved postoperative outcomes.

Therefore, the aim of this study was to translate, culturally adapt, and validate the CFS in Serbia in patients undergoing elective major surgical procedures.

## 2. Materials and Methods

### 2.1. Study Population and Design

This cross-sectional study was conducted at the Clinic of Urology, University Clinical Centre of Serbia, from July 2024 to January 2025, with the aim of validating the Clinical Frailty Scale. The study protocol complied with the Declaration of Helsinki and was approved by the Ethics Committee of the Medical University of Belgrade (No. 25/VII-1) and the University Clinical Centre of Serbia (No. 1600/54). Written informed consent was obtained from all participants.

Eligibility criteria included patients aged ≥50 years diagnosed with urological malignancy who were scheduled for a major elective surgery. Exclusion criteria were severe cognitive impairment measured by an MMSE score (MMSE 0–17), severe hearing impairment, aphasia precluding effective communication, bilateral arm weakness due to previous neurological disease or injury, no computed tomography within the previous 90 days, as CT imaging was required to calculate the Skeletal Muscle Index (SMI) or missing clinical data. Furthermore, patients who declined participation, were unable to cooperate, or could not complete the frailty assessment were also excluded.

### 2.2. Sample Size Calculation

For reliability analyses, including inter-rater and test–retest reliability assessed using the intraclass correlation coefficient (ICC), the required sample size was calculated using a precision-based approach for ICC estimation. Based on published recommendations for ICC reliability studies, the calculation was performed assuming an expected ICC of 0.85, two measurements per subject, and aiming for a 95% confidence interval with a width not exceeding 0.10. Based on these assumptions, a minimum sample size of 124 participants was required to achieve adequate precision of the reliability estimate [24,25]. In addition, concurrent validity was evaluated by examining correlations between the CFS scores and other validated frailty instruments, including the EFS and FRAIL scales. Assuming a correlation coefficient of r = 0.25, a two-sided significance level of α = 0.05, and 80% statistical power, the minimum required sample size was approximately 123 participants [26,27].

A total of 149 participants were included in the final analysis, exceeding the minimum recommended sample size for psychometric validation studies and providing sufficient statistical power and precision for both reliability and validity analyses.

### 2.3. Translation and Adaptation Procedure

**Figure 1:**
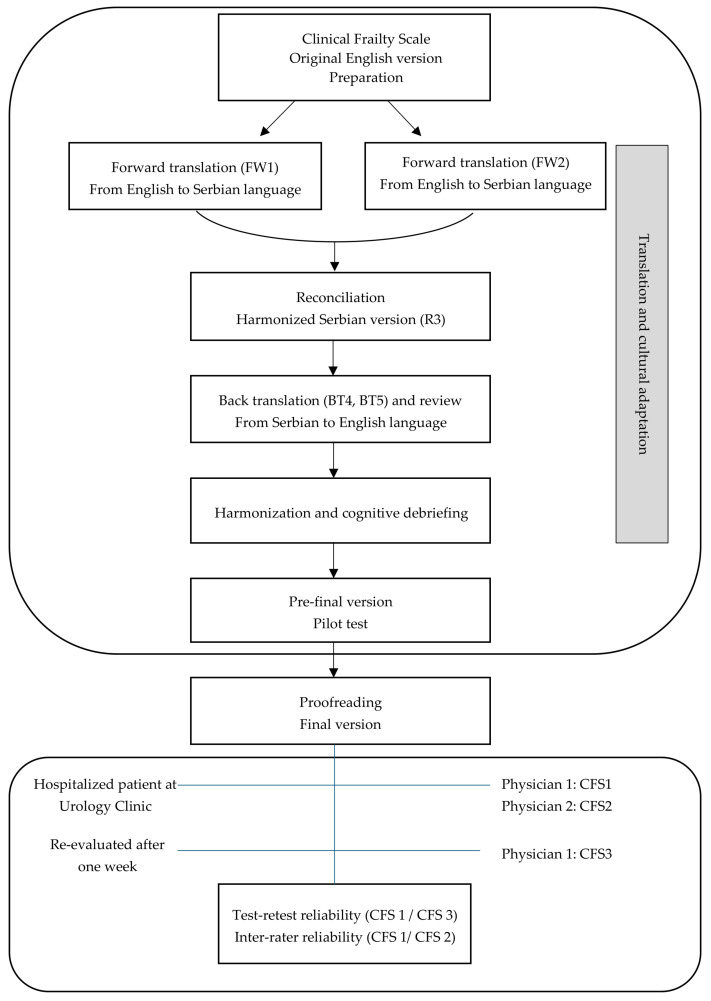
Flowchart of the Serbian translation and cross-cultural adaptation of the Clinical Frailty Scale (CFS).

The Clinical Frailty Scale (CFS) was translated into Serbian with permission from its original creators at Dalhousie University, granted on 27 May 2024 (Reference No. 20240527-01). The translation and cultural adaptation of the CFS were performed in accordance with the internationally accepted ISPOR Good Practice Guidelines [28]. Two independent forward translations from English to Serbian were produced by native Serbian speakers fluent in English and familiar with the concept of frailty. The purpose of this step was to obtain two linguistically accurate but independently developed Serbian versions of the scale. These versions were then compared and reconciled into a single Serbian version by an expert panel. This reconciled Serbian version was then back-translated into English by two independent translators blinded to the original instrument. The back-translations were compared with the original English version in order to identify potential discrepancies in meaning, terminology, or clinical interpretation. An expert team consisting of a principal investigator, two anesthesiologists with experience in perioperative medicine, an epidemiologist, and a professional philologist, reviewed all discrepancies between the original and back-translated versions and produced a pre-final Serbian version. Cognitive debriefing was then conducted with five healthcare professionals to assess clarity, comprehensibility, cultural relevance, and practical applicability of the pre-final Serbian version in clinical use. Participants were asked to comment on whether the wording was understandable, if any terms were ambiguous, and whether the descriptions were appropriate for Serbian-speaking healthcare professionals. Based on their feedback, minor linguistic adjustments were made. The final Serbian version was subsequently proofread and approved by the expert panel. (Figure 1).

### 2.4. Data Collection and Additional Measurements

All data were collected during the preoperative assessment visit. Standard demographic variables, including age, sex, Body Mass Index (BMI), educational level, marital status, place of residence, and medical history (comorbidities and medication use, defined as the number of medications), were recorded. The Charlson Comorbidity Index (CCI) was calculated using a standardized scoring method [29]. Patients were classified according to the American Society of Anesthesiologists Physical Status (ASA-PS) [30]. The Mini-Mental State Examination (MMSE) was administered to assess cognitive status [31]. Individuals with MMSE scores between 30 and 24 were considered to have no cognitive deficit, whereas those with scores ranging from 23 to 18 were identified as having mild cognitive impairment. Skeletal muscle mass was evaluated by measuring the cross-sectional muscle area at the third lumbar vertebra (L3) level using computed tomography, with adjustments for height in square meters, presented as the Skeletal Muscle Index (SMI). The cutoff value for low muscle mass was set at 52.3 cm^2^/m^2^ for men or 38.6 cm^2^/m^2^ for women who were not obese (BMI < 30 kg/m^2^) and 54.3 cm^2^/m^2^ for men or 46.6 cm^2^/m^2^ for women who were obese (BMI ≥ 30 kg/m^2^) [32,33,34]. Muscle strength was evaluated by measuring hand grip strength (HGS) using a Jamar hydraulic hand dynamometer (Patterson Medical, Ltd. Nottinghamshire, UK), with threshold values set according to the European Working Group on Sarcopenia in Older People (EWGSOP) criteria (male < 30 kg and female < 20 kg) [35]. Muscle performance was evaluated through a gait speed test, calculated by dividing the distance of 4 m by the time required (m/s) to walk this distance, with a cutoff value of 0.8 m/s [32].

### 2.5. Preoperative Frailty Assessment

Frailty was assessed preoperatively by assessing the baseline functional level using three different tools: the Serbian-translated version of the Clinical Frailty Scale (CFS), the Edmonton Frail Scale (EFS) [36], and the Fatigue, Resistance, Ambulation, Illness, and Loss of Weight (FRAIL) scale [37,38].

The Clinical Frailty Scale (CFS) is a valid and reliable tool for assessing frailty, developed for the Canadian Study of Health and Aging [12]. Initially developed as a 7-point scale, the tool has been updated to a 9-point version. The CFS combines clinical judgment with objective assessment of fitness and frailty, considering factors such as comorbidities, cognitive function, and functional decline. Patients are rated on a scale from very fit (1) to terminally ill (9). Scores ranging from 1 to 3 indicate non-frailty, while scores of 4 and higher denote frailty. The Edmonton Frail Scale (EFS) is another frequently used frailty tool in clinical settings. The EFS scores are associated with adverse surgical outcomes, including increased postoperative complications and prolonged hospital stay [9,39,40]. This multidimensional performance-based tool evaluates physical performance, cognition, social support, and general health. It comprises 11 questions across nine domains, with scores ranging from 0 to 17. Patients were categorized according to the standard bedside version as fit (0–3), vulnerable (4–5), mildly frail (6–7), moderately frail (8–9), or severely frail (≥10) [41,42]. The Serbian version of the scale is available online from the official author [42]. The Fatigue, Resistance, Ambulation, Illness, and Loss of Weight (FRAIL) scale is a self-reported instrument for evaluating frailty by both healthcare and non-healthcare professionals. It evaluates the presence or absence of five dimensions: fatigue, resistance, ambulation, illness, and weight loss, with scores ranging from 0 to 5 points (0 indicating robustness, 1–2 indicating pre-frailty, and 3–5 indicating frailty) [10].

The CFS was scored preoperatively for each patient by the first examiner, a resident of anesthesiology (CFS1), and by the second examiner, an anesthesiology specialist (CFS2), who were blinded to each other’s evaluations. To evaluate test–retest reliability, the CFS was reassessed by the initial examiner after a 7-day interval (CFS3). All tests were performed to assess the baseline function of patients that they had before hospitalization. One of the three research investigators, who was blinded to the CFS evaluation, performed the EFS and FRAIL assessments at any later point during the preoperative period. Before the start of the study, all three researchers underwent structured training on administering the CFS and EFS. The frailty score assessed by an anesthesiology specialist (CFS2) was used as the reference CFS value in the analysis.

Construct validity was evaluated by examining the relationship between CFS scores and selected sociodemographic and clinical characteristics previously reported to be associated with frailty. Based on this hypothesis, it was expected that older age, higher Charlson Comorbidity Index (CCI), higher (ASA-PS) status, falls in the previous months, sensory function disorders (e.g., hearing impairment), decreased muscle mass, decreased function and performance, and cognitive impairment would be associated with the presence of frailty (higher CFS scores).

### 2.6. Statistical Analysis

All data were analyzed using SPSS Statistics Version 29.0.2.0. To assess the distribution of the evaluated continuous variables, the Kolmogorov–Smirnov test was used. Descriptive data are presented as frequencies and percentages (%) for categorical variables and means and standard deviations (SDs) for continuous variables. The instrument’s reliability was evaluated by assessing internal consistency through Cronbach’s α coefficient and by determining inter-rater and test–retest reliability using the intraclass correlation coefficient (ICC) model with a 95% confidence interval. The levels of reliability, as determined by the ICC test, were categorized as poor (<0.50), moderate (0.50–0.74), good (0.75–0.90), and excellent (>0.90) [43]. To assess criterion-concurrent validity, the CFS was evaluated against the EFS and FRAIL scores using Spearman’s correlation coefficient (ρ). The interpretation of the correlation values was categorized as follows: a value below 0.20 suggested a negligible association; a range of 0.21 to 0.40 indicated a weak association; 0.41 to 0.60 reflected a moderate association; 0.61 to 0.80 showed a strong association; and values exceeding 0.81 demonstrated a very strong association [44]. Construct validity was evaluated using known-group comparison to evaluate how well the CFS discriminates between subgroups of the study sample that differed in age, ASA status, cognitive impairment, sensory impairment, balance, muscle strength, and performance. For comparison between groups, the test for trends was used. For all analyses, *p* < 0.05 was considered statistically significant.

### 2.7. Ethical Considerations

Approval for the study was obtained from the Ethics Committee of the Faculty of Medicine at the University of Belgrade, Serbia (decision number 25/VII-1, dated 8 July 2024) and the Ethics Committee of the University Clinical Centre of Serbia (1600/54). Written informed consent was obtained from all participants in accordance with the study protocol and the ethical guidelines of the Declaration of Helsinki.

## 3. Results

### 3.1. Translation, Cross-Cultural Adaptation and Linguistic Validation

**Figure 2:**
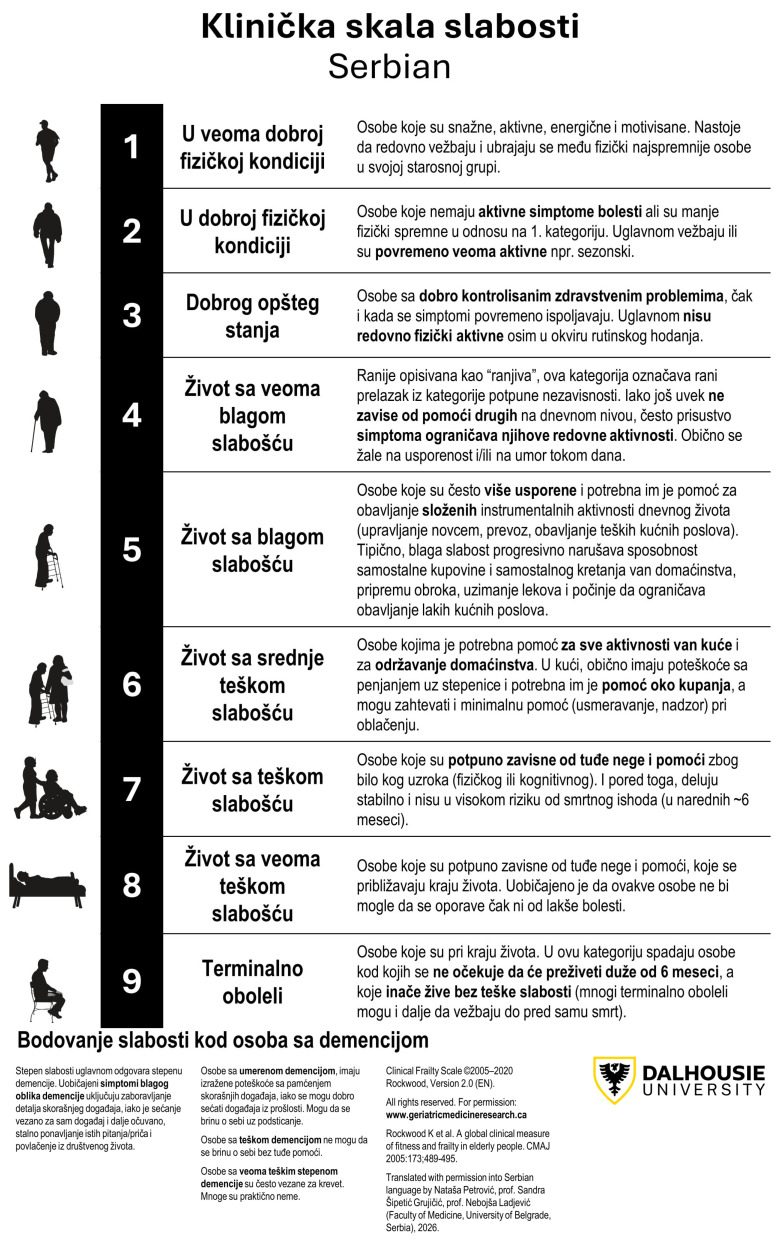
Clinical Frailty Scale in the Serbian language [45].

The process of translation and linguistic validation is summarized in Figure 1, and the Serbian translation of the Clinical Frailty Scale (CFS-9 2.0 Serbian) is shown in Figure 2. Overall, the forward translations showed a high level of agreement in both interpretation and wording, and the back-translation was conceptually consistent with the original English version. During the cognitive debriefing, the participants demonstrated good understanding and cultural appropriateness of the Serbian version. Only minor linguistic adjustments were required, mainly to improve clarity, readability, and grammatical accuracy, while preserving the conceptual meaning of the original CFS items. No major conceptual or cultural modifications were necessary.

### 3.2. Descriptive Statistics

During the study period, 216 patients were initially screened for eligibility, with 149 patients meeting all inclusion criteria and subsequently included in the final analysis. The mean age of the participants was 66.34 ± 7.52 years, and 114 (77%) were male. Most participants were married (77%) and lived in urban areas (75%). A college or university degree was attained by 32% of the participants. The mean BMI was 26.8 ± 5.0 kg/m^2^. The majority of participants presented one or more comorbidities (91.9%), with almost three-quarters of the study population (73.8%) having severe comorbidities (Charlson Comorbidity Index ≥ 5). Thirty-four patients were categorized as frail according to the CFS values (22.8%). Patient’s characteristics are presented in Table 1.

For the frailty assessment, CFS scores ranged from 1 to 5. The mean CFS score assessed by the first investigator, an anesthesiology resident at the initial assessment (CFS1), was 2.91 ± 0.903, while the mean score assessed by the second investigator, an anesthesiology specialist (CFS2), was 2.99 ± 0.951. The repeated assessment performed by the first investigator after 7 days (CFS3) showed a mean score of 2.98 ± 0.911. (Table 2). The most prevalent CFS score was 3—“ Managing Well”—assessed in 66 patients (44.3%), followed by 2—“ Fit”—assessed in 47 patients (31.5%) (Table 2).

The Kolmogorov–Smirnov test indicated that the distribution of CFS scores significantly deviated from the normal distribution (*p* < 0.001 for all three estimates).

### 3.3. Inter-Rater Reliability, Test–Retest Reliability and Convergent Validity

The inter-rater reliability between CFS1 and CFS2 was excellent, with an intraclass correlation coefficient (ICC) of 0.957 (95% CI 0.941–0.968; *p* < 0.001). Internal consistency between the two measurements was also very high (Cronbach’s α = 0.978). The test–retest reliability assessed by comparing the first and repeated measurements, performed after a one-week interval by the same investigator (CFS1 vs. CFS3), showed similarly high stability, with an ICC of 0.958 (95% CI 0.943–0.970; *p* < 0.001) and Cronbach’s = 0.979, indicating excellent reliability of the repeated measurement.

For further analysis, CFS2 scores, assessed by the anesthesiology specialist, were used as the reference CFS value. Convergent validity analysis demonstrated a significant positive correlation between the Clinical Frailty Scale (CFS) and the Edmonton Frail Scale (EFS) (ρ = 0.698, *p* < 0.001), indicating a strong association between these two instruments for assessing frailty. A significant correlation was also observed between the CFS and the FRAIL scale (ρ = 0.614, *p* < 0.001) (Table 3).

### 3.4. Construct Validity

In the known-group analysis, the CFS was able to effectively identify differences among various subgroups of individuals based on clinically relevant characteristics such as age, ASA physical status, cognitive and sensory impairments, balance, muscle strength, and performance. As expected, those who were older, with higher ASA-PS scores, had several comorbid conditions with higher Charlson Comorbidity Index; diminished muscle strength, mass, and performance; impaired mobility; and cognitive impairment, with sensory impairment, as well as showing higher CFS scores. The statistically significant differences in CFS scores across these subgroups confirmed the expected relationships and supported the construct validity of the instruments. However, no statistically significant association was observed between CFS scores and Skeletal Muscle Index (SMI) (Table 4).

## 4. Discussion

This study reports the development and validation of the Serbian version of the Clinical Frailty Scale (CFS) and represents the first psychometric evaluation of this instrument in Serbia. The availability of a culturally adapted and linguistically validated frailty assessment tool is an important step toward improving the identification and clinical management of frailty in Serbia, where standardized instruments of frailty assessment have been limited. In addition, the Serbian language is widely understood across several countries in the Balkans and among other South Slavic populations, which may facilitate broader regional application of the Serbian version of the CFS and support its use in future multicenter research in this linguistic area.

Translation and linguistic validation were conducted following the ISPOR guidelines [28]. Experts from various fields carried out the translation, addressing the multidisciplinary aspect of the CFS. In our study, patients were divided into two categories: frail and non-frail. Those previously labeled as “Vulnerable” with a CFS score of 4 are in our study, according to the updated CFS 9.0 version, classified as “Living with mild frailty” [46]. Consequently, in our study, 22.8% of patients had a CFS score of 4 or higher and were classified as frail.

The Serbian version of the Clinical Frailty Scale (CFS) demonstrated excellent inter-rater and test–retest reliability, as indicated by the intraclass correlation coefficient (ICC). Although the CFS is an ordinal categorical scale, it is frequently treated as a quasi-continuous variable in psychometric validation research. Consequently, the ICC is commonly utilized to assess reliability across numerous studies, allowing comparison with previously published data [14,16,21,22,47,48]. In our study, the ICC between the two evaluators was 0.957 (95% CI 0.941–0.968), indicating a high level of agreement between the anesthesiology specialist and the resident. After a one-week period, the ICC for test–retest reliability was 0.958 (95% CI 0.943–0.970), reflecting significant short-term consistency and excellent reliability in repeated measurements. This aligns with validation studies conducted in other languages. In a French multinational prospective study conducted among very old intensive care (ICU) patients, Abraham et al. reported inter-rater ICC values of 0.76 between nurses and 0.73 between a doctor and a nurse for the French version [21]. Another validation study among non-ICU patients, a Greek validation study, also showed excellent inter-observer reliability, with an inter-rater ICC value of 0.87 and a test–retest ICC of 0.89 [16]. A Danish multicenter study found ICC values ranging from 0.81 to 0.90 across various healthcare professionals, and the Swedish version showed excellent agreement among 12 raters with an ICC value of 0.969; however, the generalizability of these results might be limited due to the use of case vignettes instead of real patients [22,23]. The higher ICC observed in our cohort (0.957) compared to other validations may be attributed to the relatively homogeneous nature of the surgical patient population, which is consistent with the findings of Chang et al. [47]. They demonstrated excellent inter-rater reliability and test–retest reliability (ICC = 0.966) among older adults with end-stage knee osteoarthritis preoperatively. In contrast, more heterogeneous populations, as seen in other studies, might result in greater variability and lower reliability. In our study, we confirmed the high reliability of the Serbian version of the CFS, emphasizing its reproducibility and consistency, which ensures that various evaluators can obtain similar scores in preoperative assessments.

The validity of the Serbian CFS was assessed by examining its convergent and construct validity. The Kolmogorov–Smirnov test indicated that CFS scores significantly deviated from the normal distribution (*p* < 0.001 for all three estimates). This finding was taken into account in the selection of statistical procedures. Therefore, non-parametric methods were used where appropriate, including Spearman’s rank correlation for convergent validity and non-parametric tests for group comparisons. Thus, the lack of normality did not compromise the validity analyses but informed the use of statistical methods suitable for ordinal and non-normally distributed data. Convergent validity was confirmed through a strong positive correlation between both the EFS (ρ = 0.698) and the FRAIL scale (ρ = 0.614), suggesting a significant agreement between these instruments in evaluating frailty. These findings are consistent with the existing literature. Jesadaporn et al. reported a moderate correlation between the Thai version of the CFS and the FRAIL scale (ρ = 0.53) [19], whereas the Chinese and Korean versions demonstrated very strong positive correlations with the FRAIL scale (ρ = 0.802 and ρ = 0.80, respectively) [18,47]. These differences may be partly explained by variations in the study population. The Korean and Chinese cohorts included older, frailer patients, whereas our cohort, similar to the Thai study population, consisted of relatively younger patients, with no participants classified in categories 8 and 9. Another possible explanation is related to the self-reported nature of the FRAIL scale. The lower degree of correlation with the FRAIL scale can be explained by the presence of social desirability bias, where patients, fearing delays in surgical intervention, tend to subjectively present their health condition as better than it objectively is. In a study on the utility of the CFS in patients undergoing vascular surgery, there was a moderate level of agreement between the EFS and CFSs (87.6–89.7% agreement) [49], whereas other studies confirmed a strong correlation with the EFS (ρ = 0.755 0.85) [50,51]. This correlation data supports the validity of the Serbian version of the CFS.

In an analysis comparing known groups, the CFS was able to effectively identify differences among various subgroups of individuals based on factors such as age, ASA physical status, cognitive and mild-to-moderate sensory impairments, balance, muscle strength, and performance. As expected, those who were older had multiple comorbidities, reduced muscle strength, impaired mobility and balance, sensory impairment, or mild cognitive deficits, which showed higher CFS scores. The statistically significant differences in CFS scores across these subgroups confirmed the expected relationships and supported the construct validity of the instruments. The association between CFS scores and cognitive impairment and sarcopenia has been previously reported, and our results are consistent with earlier studies [12,16,18]. In our study, no significant correlation was identified between the CFS score and muscle mass, as represented by the Skeletal Muscle Index (SMI). This represents an important finding, as it suggests that frailty assessed by the CFS may reflect a broader clinical and functional vulnerability rather than muscle mass alone. In contrast to SMI, clinical markers such as reduced handgrip strength and impaired gait speed were more closely associated with frailty. These findings align with those of previous studies, which reported that low muscle mass is more prevalent in sarcopenic patients, whereas low handgrip strength and reduced gait speed are more commonly observed in frail patients [17,52]. Therefore, the absence of a significant association between the CFS and the SMI supports the concept that frailty and sarcopenia, although sharing some similarities, are two distinct conditions [52]. Given that these important clinical characteristics can be rapidly identified using the CFS, the scale represents a practical and valuable instrument for frailty screening.

The strengths of the study are reflected in the translation and adaptation of the CFS strictly according to ISPOR guidelines, with a back-translation that guarantees linguistic equivalence. The obtained ICCs (>0.95) show that the scale is extremely stable regardless of the assessor (specialist or resident). The inclusion of parameters such as SMI (from CT findings), hand grip strength, and gait speed adds more depth to construct validation.

This study has a few limitations that should be acknowledged. First, this study was conducted at a single tertiary-care institution and included a specific clinical population of patients undergoing major urological oncological surgery. Therefore, the findings may not be fully generalizable to other healthcare settings, i.e., primary care. Additionally, there were no severe frail and/or terminally ill patients (CFS 6–9) in this study sample; therefore, the scale was not tested in this spectrum. This limitation is likely related to the elective surgical setting, in which patients with advanced frailty are less frequently selected for major operative treatment. Finally, further validation of the Serbian version of the CFS in larger, multicenter cohorts and in different clinical environments is needed to confirm the external validity and broader applicability of the Serbian version of the CFS.

## 5. Conclusions

The Serbian version of the CFS showed excellent psychometric properties. High inter-rater and test–retest reliability has been demonstrated, along with a strong correlation with the EFS and FRAIL scales. The scale successfully discriminates against patients according to age and comorbidities. The study results confirm that the CFS presents a reliable, valid, and practical tool that can be used for rapid preoperative identification of frailty syndrome in clinical practice in Serbia and the wider South Slavic-speaking area.

## Figures and Tables

**Table 1:** Baseline characteristics of the study participants (*n* = 149).

Baseline Characteristics	Overall (*n* = 149)
**Demographic Data**	
Age (years), Mean (SD)	66.34 (7.52)
Male, *n* (%)	114 (76.5)
Married, *n* (%)	115 (77.2)
Place of Residence, *n* (%)	
Urban	111 (74.5)
Rural	38 (25.5)
Education, *n* (%)	
Primary or Secondary School	101 (67.8)
College or University Degree	48 (32.2)
BMI (kg/m^2^), Mean (SD)	26.8 (4.67)
**Clinical Data**	
Presence of Comorbidities, n (%)	137 (91.9)
HTA	126 (84.6)
COPD	21 (14.1)
DM	37 (24.8)
Charlson Comorbidity Index (CCI), Mean (SD)	5.70 (1.98)
Number of Medications, Mean (SD)	3.62 (2.52)
ASA, *n* (%)	
1–2	86 (57.7)
3–4	63 (42.3)
Degree of Cognitive Impairment ^a^, *n* (%)	
No Cognitive Impairment	142 (95.3)
Mild–Moderate Cognitive Impairment	7 (4.7)
Hand Grip Strength (kg), Mean (SD)	39.32 (11.55)
SMI (kg/m^2^), Mean (SD)	48.13 (9.78)
**Frailty Assessment**	
CFS, *n* (%)	
1	2 (1.3)
2	47 (31.5)
3	66 (44.3)
4	19 (12.8)
5	15 (10.1)
CFS Group ^b^, *n* (%)	
Frail	34 (22.8)
Non Frail	115 (77.2)

Abbreviation: SD, standard deviation; BMI, Body Mass Index; HTN, arterial hypertension; COPD, chronic obstructive pulmonary disease; DM, diabetes mellitus; CFS, Clinical Frailty Scale Score; MMSE, Mini-Mental State Examination; SMI, Skeletal Muscle Index, adjusted for square meter of height. ^a^ no cognitive impairment: MMSE = 30–24; mild–moderate cognitive impairments: MMSE = 23–18. ^b^ non-frail: CFS2 = 1–3; Frail: CFS2 ≥ 4.

**Table 2:** Descriptive statistics of Clinical Frailty Scale (CFS) scores across assessments.

Assessment	*n*	Min	Max	Mean	SD
CFS1	149	1	5	2.91	0.903
CFS2	149	1	5	2.99	0.951
CFS3	149	2	5	2.98	0.911
Total	149				

Abbreviations: CFS1—CFS assessed by first investigator (anesthesiology resident); CFS2—CFS score assessed by second investigator (anesthesiology specialist); CFS3—repeated CFS assessment after 7 days by first investigator (anesthesiology resident).

**Table 3:** Reliability and validity tests of the CFS.

Type of Reliability	Variables	Statistical Method	Value	95% CI	*p* Value
Inter-rater reliability	CFS1 vs. CFS2	ICC	0.957	0.941–0.968	<0.001
Test–retest reliability	CFS1 vs. CFS3	ICC	0.958	0.943–0.970	<0.001
Convergent validity	CFS * vs. EFS	Spearman coefficient	0.698		<0.001
Convergent validity	CFS * vs. FRAIL	Spearman coefficient	0.614		<0.001

Abbreviations: CFS1, clinical frailty score assessed by first examiner, anesthesiology resident; CFS2, clinical frailty score assessed by second examiner, anesthesiology specialist; CFS3, re-evaluation of clinical frailty score by first examiner; ICC, the intraclass correlation coefficient (two-way mixed effects, consistency); CFS, Clinical Frailty Scale; EFS, Edmonton Frail Scale; FRAIL, Fatigue, Resistance, Ambulation, Illness, and Loss of Weight (FRAIL) scale. * CFS2 value was used for the convergent validity correlations.

**Table 4:** Comparison of CFS scores across sociodemographic and health-related characteristics.

	*n*	CFS Score (Mean ± SD)	*p* Value
**Age Group (years)**			
50–64	55	2.64 ± 0.70	*p* ≤ 0.001
65–74	75	3.01 ± 0.94	
>75	19	3.89 ± 1.04	
**ASA-PS**			
1–2	86	2.64 ± 0.80	*p* ≤ 0.001
3–4	63	3.46 ± 0.95	
**Multimorbidity (CCI)**			
2–3	15	2.40 ± 0.51	*p* ≤ 0.001
4–5	98	2.81 ± 0.88	
6–7	20	3.65 ± 0.93	
≥8	16	3.81 ± 0.83	
**Polypharmacy (≥5 medications)**			
No	102	2.65 ± 0.75	*p* ≤ 0.001
Yes	47	3.72 ± 0.93	
**Degree of Cognitive Impairment (MMSE)**			
No cognitive impairment	142	2.92 ± 0.91	*p* ≤ 0.001
Mild cognitive impairment	7	4.29 ± 0.95	
**Falls in the Previous Year**			
No	140	2.92 ± 0.92	*p* ≤ 0.001
Yes	9	4.00 ± 0.87	
**Sensory Hearing Impairment**			
No	145	2.94 ± 0.92	*p* ≤ 0.001
Yes	4	4.50 ± 1.00	
**Hand Grip Strength**			
Normal	141	2.91 ± 0.89	*p* ≤ 0.001
Low	8	4.25 ± 1.17	
**Skeletal Muscle Index (SMI) ^a^**			
Normal	57	2.91 ± 0.87	*p* = 0.460
Low	92	3.03 ± 1.00	
**Gait Speed**			
>0.8 m/s	96	2.56 ± 0.63	*p* ≤ 0.001
≤0.8 m/s	53	3.75 ± 0.96	

Abbreviations: ASA-PS, American Society of Anesthesiologists Physical Status; CCI, Charlson Comorbidity Index; MMSE, Mini-Mental State Exam; SMI, Skeletal Muscle Index, adjusted for square meter of height. ^a^ calculated from cross-sectional skeletal muscle area at the L3 level on computed tomography (CT) images.

## Data Availability

The raw data supporting the conclusions of this article will be made available by the authors on request.

## Abbreviations

The following abbreviations are used in this manuscript:

BMI	Body Mass Index
CFS	Clinical Frailty Scale
EFS	Edmonton Frail Scale
FRAIL	Fatigue, Resistance, Ambulation, Illnesses, and Loss of Weight
ICC	Intraclass Correlation Coefficient
SD	Standard Deviation
SMI	Skeletal Muscle Index
ASA-PS	American Society of Anesthesiologists Physical Status
CCI	Charlson Comorbidity Index
MMSE	Mini-Mental State Exam
HTN	Arterial Hypertension
COPD	Chronic Obstructive Pulmonary Disease
DM	Diabetes Mellitus
EWGSOP	European Working Group on Sarcopenia in Older People
